# Development and matching of binocular orientation preference in mouse V1

**DOI:** 10.3389/fnsys.2014.00128

**Published:** 2014-07-24

**Authors:** Basabi Bhaumik, Nishal P. Shah

**Affiliations:** Electrical Engineering Department, Indian Institute of Technology DelhiNew Delhi, India

**Keywords:** mouse V1, critical period for orientation matching, receptive field alignment, orientation selectivity, orientation map

## Abstract

Eye-specific thalamic inputs converge in the primary visual cortex (V1) and form the basis of binocular vision. For normal binocular perceptions, such as depth and stereopsis, binocularly matched orientation preference between the two eyes is required. A critical period of binocular matching of orientation preference in mice during normal development is reported in literature. Using a reaction diffusion model we present the development of RF and orientation selectivity in mouse V1 and investigate the binocular orientation preference matching during the critical period. At the onset of the critical period the preferred orientations of the modeled cells are mostly mismatched in the two eyes and the mismatch decreases and reaches levels reported in juvenile mouse by the end of the critical period. At the end of critical period 39% of cells in binocular zone in our model cortex is orientation selective. In literature around 40% cortical cells are reported as orientation selective in mouse V1. The starting and the closing time for critical period determine the orientation preference alignment between the two eyes and orientation tuning in cortical cells. The absence of near neighbor interaction among cortical cells during the development of thalamo-cortical wiring causes a salt and pepper organization in the orientation preference map in mice. It also results in much lower % of orientation selective cells in mice as compared to ferrets and cats having organized orientation maps with pinwheels.

## Introduction

In the visual system, signals from the left and the right eyes first converge in the primary visual cortex, V1. Precise and selective connections between neurons are established during developmental processes for proper functioning of the nervous system. In simple cells in V1, the spatial layout of ON and OFF subregions determines the orientation selectivity of simple cells (Hubel and Wiesel, 1962). For normal binocular perceptions, such as depth and stereopsis, binocularly matched orientation preference between the two eyes is required. Little is known about the developmental process for binocularly matched orientation preference between the two eyes. Though cortical cells mostly have binocularly matched orientation (OR) preference between the two eyes in cats and monkeys, cells with interocular difference in preferred OR (IDPO) are also reported. Blakemore et al. (1972) have reported a range a of ±15° (*S* = 6–9°) IDPOs in cat. Bridge et al. (2001) have reported a range of ±20° (*S* = 9.22°) IDPOs in macaque. The binocularly matched orientation preference is established in mice after eye opening. Thus, study on mice provides a unique opportunity to investigate development of binocularly matched orientation preference between the two eyes (Wang et al., 2010; Sarnaik et al., 2014) during normal development.

Neurons in mouse V1 are selective for stimulus orientation (Dräger, 1975; Metin et al., 1988; Van Hooser, 2007; Niell and Stryker, 2008). The development of orientation selectivity in V1 has been extensively studied in cats, ferrets, and monkeys (Hubel and Wiesel, 1963, 1968; Chapman and Stryker, 1993; Sato et al., 1996; Ringach et al., 1997; White et al., 2001) in contrast to mice. Unlike mammals with frontally placed eyes, mice have more laterally placed eyes. Also in the binocular visual cortical area the retinal inputs from contralateral and ipsilateral eyes are not roughly equal as in cats but the ratio of contralateral-to-ipsilateral responses in binocular V1 is approximately 2:1 (Coleman et al., 2009). Experimental studies (Dräger, 1975; Mangini and Pearlman, 1980; Metin et al., 1988; Frenkel et al., 2006; Niell and Stryker, 2008) suggest that in mice the visual cortical neurons respond to orientation stimulus similarly to that in cat (Tan et al., 2011) and monkey (Van den Bergh et al., 2010).

There is a scarcity of models on development of orientation selectivity in mice. Recently two computational models (Hansel and van Vreeswijk, 2012; Roy et al., 2013) were proposed on orientation selective response in layer 2/3 neurons. In both the models orientation selectivity was studied in layer 2/3 with feedforward input from layer IV neurons and lateral connections within layer 2/3. Layer IV neurons are modeled with Gabor filters and have salt-and-pepper organization of orientation selectivity. At present no model exists for receptive field development in mouse V1, nor does a model exist that captures a salt-and-pepper organization of orientation selectivity.

Since the seminal work of Turing (1952) reaction diffusion equations have been applied to biological pattern formations extensively (Murray, 1991). Reaction-diffusion equation comprises a reaction term and a diffusion term. In this paper we extend and apply our earlier reaction-diffusion based model (Bhaumik and Mathur, 2003; Siddiqui and Bhaumik, 2011) to model responses in mouse V1. The reaction term in our model captures competition for resources available at (i) the pre-synaptic cell, and (ii) the post-synaptic cell. The diffusion cooperation between neighboring cells are modeled through the diffusion term. We report the following.

- *RF development of cortical cells in monocular and binocular region in mouse V1*: In our model cortex 39% cells are orientation selective in binocular region and 38% in monocular region. Reported median orientation selectivity index (OSI) value in mouse is 0.31 (Tan et al., 2011). For our modeled cells with *OSI* > 0.3, the mean OSIs in the two regions are 0.46 and 0.41, respectively. The corresponding orientation tuning mean half widths at half height (HWHH) in binocular and monocular regions are respectively, 36° and 39.4°. The mean spatial frequency in binocular zone is 0.038 cycles/° for cells with *OSI* > 0.3.
- *Orientation (OR) preference and ocular dominance (OD) maps:* In both monocular and binocular region we find lack of clustering of similar orientation preferences i.e., salt-and-pepper organization (Dräger, 1975; Mangini and Pearlman, 1980; Metin et al., 1988; Bonin et al., 2011) in OR preference map. In the binocular V1, neurons are usually contralateral eye dominated in mice (Dräger, 1975, 1978). Wang et al. (2010) has reported contralateral bias in OD with a mean OD of 0.19 ± 0.03. The average OD in our model cortex is −0.1031. The negative average OD indicates that cells in the model binocular V1 have contralateral eye bias. The OD map is unstructured.
- *The critical period:* We show how the starting and the closing time of the critical period affects orientation tuning, subfield matching in RFs, and alignment of orientation preference in cortical cells between the two eyes in the binocular region in mouse V1.

## Methods

### Three layer visual pathway model

**Figure 1:**
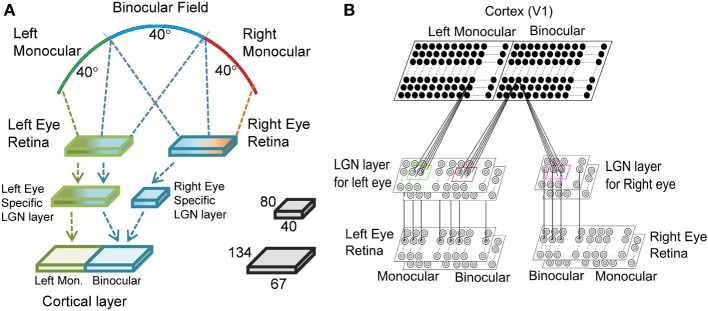
**The three layered visual pathway model**. We have modeled 40° of binocular and left monocular visual fields each as shown in **(A)**. The left and the right eye retinae are 80 × 80 each. The left eye specific LGN layer is 80 × 80 with 40 × 80 region getting input from left monocular field and the rest 40 × 80 region from binocular field of vision. The right eye specific LGN layer is 40 × 80 and receives input from binocular field of vision. The cortical layer in the right hemisphere has left monocular and binocular regions of size 67 × 134 each. **(B)** The LGN layer for each eye has two sheets of cells each with center-surround structure—one for ON center and another for OFF center type cells. Each cortical cell in the left monocular region gets input from a 13 × 13 section of cells from the left eye specific LGN layer, whereas each cell in binocular region gets input from a 13 × 13 section of cells from both the left eye and the right eye specific LGN layers.

Mouse V1 has two zones: (i) the monocular zone, where neurons receive inputs only from the contralateral eye, and (ii) the binocular zone, where neurons receive inputs from both ipsi- and contralateral eyes as shown in Figure 1A. To obtain responses of cortical cells in our model mouse V1 we have used a three-layer visual pathway model as depicted in Figure 1B. In mouse the dendrites of retinal ganglion cells (RGCs) in the inner plexiform layer (IPL) of retina are separated into ON or OFF sublamina (Tian, 2004). We have modeled ON of OFF RGC as two separate layers. Retina for left (contralateral) eye is modeled with two 2D 80 × 80 sheet of ON and OFF center ganglion cells lying one over the other. The right (ipsilateral) retina is modeled with two 2D 40 × 80 layer of ON and OFF center ganglion cells. Mouse RGCs have center-surround receptive field structure with center fields having a radius of 5.5° and the surround field radius is 16.98° as reported in Grubb and Thompson (2003). Center-to-center spacing between the cells is 52′ of the visual angle. The ganglion cell model used earlier (Wehmeier et al., 1989; Wörgotter and Koch, 1991; Somers et al., 1995; Bhaumik and Mathur, 2003) for cats is modified to produce realistic temporal response to visual stimuli in mice. The details are given in the Supplementary Material.

The second layer models LGN. The left and the right eye specific LGN layers are made up of 2D 80 × 80 size and 40 × 80 size sheets of LGN cells, respectively. In mouse dLGN ON- and OFF-center cells appear intermingled (Grubb et al., 2003) and unlike in retina do not form sublamina. It is reasonable to assume that initially a cortical simple cell receives inputs from equal number of ON- and OFF-type LGN cells. We have shown ON- and OFF- as two layers for ease of representation. It is reported that in cats each LGN cell receives strong input from 1 to 3 retinal cells (Chen and Regehr, 2000; Jaubert-Miazza et al., 2005). In mice about three contralateral RGCs connect to one LGN cell whereas one ipsilateral RGC connects to one LGN cell (Coleman et al., 2009). In our model we have assumed that each LGN cell receive input from one retinal cell. The normalization constant in Wörgotter and Koch's (1991) model was chosen such that LGN cells firing rate matched experimental values (Grubb and Thompson, 2004) for a 90% contrast sinusoidal grating input to retina. The firing rate for LGN cell at the 90% contrast is 36 spikes/s.

The third layer models cortical layer IV of mouse V1. Third layer is comprised of a 2D 67 × 134 size sheet of left monocular cortical cells and a 2D 67 × 134 size sheet of binocular cortical cells. In the monocular zone each cortical cell receives synaptic connections from 13 × 13 left eye specific ON/OFF LGN regions centered at its retinotopic position. In the binocular zone each cortical cell receives synaptic connections from 13 × 13 left and right eye specific ON/OFF LGN regions. Thalamic projection from 13 × 13 LGN cells defines the RF of a cortical cell and corresponds to inputs from approximately 21° in visual space. The median RF size is 21.3° in the visual space for simple cells in mice (Van den Bergh et al., 2010). In the model cortex, binocular zone covers 40° binocular visual space and monocular zone covers 40° left monocular visual space as shown in Figure 1A.

### Cortical cell model

Membrane potential of the cortical cell is evaluated using the equation (Gerstner, 1998; Bhaumik and Mathur, 2003).

$${\text{u}}_{\text{I}}\left(\text{t}\right)=\eta \left(\text{t}-{\text{t}}_{\text{fc}}\right)+\beta_{\text{l}}\sum_{\text{J},\text{k}}{\text{W}}_{\text{IJ}}^{\text{k}}\sum_{\text{fL}}\epsilon (\text{t}-{\text{t}}_{\text{fL}})+{\text{R}}_{\text{P}}$$

Where, u_I_(t) is the membrane potential of the cortical cell at location I at time t. t_fc_ is the time when the cell produced the most recent spike. W^k^_IJ_ is the strength of synapse between LGN cell at location J from cortical cell at location I. k is either left (l) or right (r). If the weight is positive, input spike train for that location is read from ON center LGN, otherwise from OFF center LGN. ε(t - t_fL_) is the EPSP (excitatory post-synaptic potential) generated in the cortical cell when there is a spike in a connected LGN cell at time t_fL_. The post-synaptic potential is scaled by the weight of the connection between LGN and cortical cell. The spikes occurring in past 90 ms are only summed up. Similarly, η(t - t_fc_) function is the refractory voltage of the cortical cell after it fires a spike at time t_fc_. R_p_ is the resting potential of the cortical cell (−70 mV). Whenever, *u*_*I*_(*t*) exceeds threshold, a spike is generated in the cortical cell and the output is 1, otherwise it is 0.

The time constants used for $\varepsilon \;\left[\varepsilon \left(\text{t}-t_{fL}\right)=Fe^{\frac{t-t_{fL}}{\tau_{rise}}}-e^{\frac{t-t_{fL}}{\tau_{decay}}}\right]$ are 5.65 ms for rise time, 2.96 ms for decay time and *F* = 1.35 in mouse compared to 12 ms rise time, 5.95 ms decay time and *F* = 1.25 in cat. Also, the time constant associated with η is 9.69 ms in mouse compared to 30 ms in cat. The time constants for mouse are smaller as compared to cat as the optimal temporal frequency for mouse is higher (4 Hz) compared to cat (0.4 Hz).

We have used a modified (Bhaumik and Mathur, 2003) Spike Response Model (SRM) (Gerstner, 1998) to incorporate synaptic scaling factor β. β is calculated as (Bhaumik and Mathur, 2003)

$$\beta =\left(H-L\right)f\left(A_{v}\right)+L$$

Where, *f*(*A*_*v*_) = 1 − *tanh*(*G*(*A*_*v*_ − θ)) and $A_{v}=\frac{1}{T}\int_{0}^{T}\sum_{\text{J}}W_{IJ}X_{J}\left(t\right)dt$. *H* and *L* are constants and determine the range of the scaling factor. *G* and θ are constants. *A*_*v*_ is the average input the cortical cell I receives over *T*. X_J_(t) belongs to {0, 1} where, X_J_ (t) represents the spike response of LGN cell at location *J* at time *t*. So, *A*_*v*_ is related to average input spike rate and weights. For cells in monocular zone: H = 8.8, L = 3, G = 0.0061, and θ = 180 and Binocular cells: H = 10, L = 4, G = 0.0061, and θ = 100.7. Different values are used for cells in monocular and binocular zone to take care of difference in membrane potential distribution in cells in the two zones.

For cortical cells in binocular region, the synaptic scaling function, β, is calculated by taking average binocular input—the normal conditions of activation for such cells. β accounts for synaptic scaling mechanism, which acts over a relatively long time scale (over a period of several hours to days) (Turrigiano, 2008). Therefore, for response to monocular activation of the mouse retina we use β value determined from binocular response of the cell. But when β determined from the binocular activation was used for monocular activation, it resulted in a low value of the membrane potential, and consequently, very low spiking. But the experimental evidence suggests that monocular activation leads to spiking which is relatively lower compared to binocular one, but not too low. Recently, it is reported that inputs from the left and the right eye add sub-linearly in visual cortical cells (Longordo et al., 2013). Instead of a fixed threshold voltage, Th, for spiking in the cortical cell we employed a variable threshold mechanism. The variable threshold mechanism acts over the course of a single experiment, taking into account the variations in input activation over successive experiments. The variable threshold is calculated using the following expression.

$$\text{Th}=\left(\text{A}-\text{B}\right)\frac{{\text{De}}^{\left(\gamma \left({\text{A}}_{\text{v}}-\text{C}\right)\right)}-{\text{e}}^{\left(-\gamma \left({\text{A}}_{\text{v}}-\text{C}\right)\right)}}{{\text{De}}^{\left(\gamma \left({\text{A}}_{\text{v}}-\text{C}\right)\right)}{+\text{e}}^{\left(-\gamma \left({\text{A}}_{\text{v}}-\text{C}\right)\right)}}+\text{B}$$

where, A = −44.5, B = 59.5, C = 100, D = 1.25; γ = 0.0081, and the expression for A_v_ is given earlier for β calculation.

**Figure 2:**
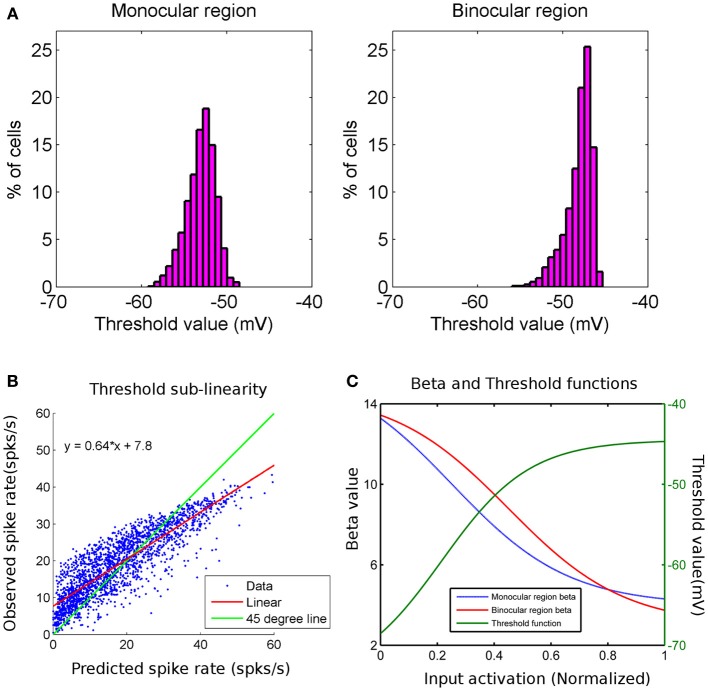
**(A)** Threshold value (mV) histograms for cortical cells in monocular (histogram on left) and binocular (histogram on right) regions are shown. The y-axis is the percentage of cells and x-axis is the threshold value in mV. **(B)** This is a scatter plot of observed binocular spike rate on y-axis and predicted binocular spike rate (as linear sum of individual left and right monocular spike rates) on x-axis for cortical cells in binocular region of cortex. The green line has a slope of 45° and the red line is the linear fit to data. The binocular spike rate is less than sum of individual monocular spike rates for higher spike rates. **(C)** Plots of β function of cells in monocular region (blue) and that in binocular region (red) (y-axis on left) with normalized input activation. β decreases as input activation increases. Plot for the threshold value (green) for all cortical cells with normalized input activation. Threshold value increases as input activation increases.

We show threshold voltage histograms in Figure 2A for cortical cells in monocular and binocular zones in the model cortex. The threshold histogram for binocular response peaks around −48 mV. This agrees with the experimentally reported threshold values (Tan et al., 2011; Figure 2). The variable threshold when incorporated in the modified SRM, captures the effect of sub-linear addition of spike rates from monocular to binocular experiments as reported in the literature (Longordo et al., 2013). In Figure 2B, the binocular response spike rate is plotted as a function of spike rate from the sum of individual monocular responses. Cell response data used in Figure 2B is the response of the cell at its preferred orientation. Data in Figure 2B is fitted with a linear function (*y* = 0.64 *x* + 7.8) and indicated with red line. The fitted linear line (shown in red) has a slope less than unity (shown as green line). At higher spike rates, the difference between the predicted spike rates from sum of monocular responses and the observed binocular spike rate is more noticeable. We also obtain spike rates in the experimentally reported range of spiking under binocular and monocular activations. A plot of β and the threshold voltage as a function of pre-synaptic input activation is shown in Figure 2C.

In the next subsection we modify our earlier thalamo-cortical synaptic weight development model (Bhaumik and Mathur, 2003; Siddiqui and Bhaumik, 2011) for cats and obtain the connections between LGN and cortical cells in mouse V1. Biologically plausible competition and cooperation principles are used to model growth and decay of thalamo-cortical synaptic strengths. Both competition (reaction) and cooperation (diffusion) involves release of neurotrophic factors. Neurotrophins are activity-dependent (Bonhoeffer, 1996; Cellerino and Maffei, 1996; Katz and Shatz, 1996; Lewin and Barde, 1996).

### Thalamo-cortical synaptic weight development: model assumptions

The model is based on the following biologically plausible assumptions.

1. *Competition for resources available at (i) the pre-synaptic cell, and (ii) the post-synaptic cell*: The number of synaptic connections a LGN cell supports is constrained by its pre-synaptic resource. Therefore, a competition exists for a pre-synaptic resource where a pre-synaptic cell has a fixed amount of resource to distribute among its axonal branches. Similarly, a post-synaptic cortical cell supports a limited number of pre-synaptic connections depending on its post-synaptic resource. A competition exists between pre-synaptic LGN axons to get connected to post-synaptic cortical cells. The LGN axons compete for neurotrophic factors released by the post-synaptic cells. Such fixed pre- and post-synaptic resources in the RGCs of gold fish (Hayes and Meyer, 1988) and optic tectum cells (Xiong et al., 1994) are reported in the literature.
2. *Diffusive cooperation between neighboring same type of ON-ON and OFF-OFF pre-synaptic LGN cells:* Experimental studies have shown that synaptic enhancement is not restricted to be specific to synapses where synchronous pre- and post-synaptic stimulation occur. But is also accompanied by spread of potentiation into other inputs on the same post-synaptic cell (Cowan et al., 1998) within a distance of approximately 70 μm (Engert and Bonhoeffer, 1997).

The existence of both pre- and post-synaptic spread of potentiation are reported in hippocampal slice CA1 (Bonhoeffer et al., 1989; Bi and Poo, 2001) and hippocampal slice culture (Muller et al., 1995; Engert and Bonhoeffer, 1997). For thalamo-cortical synaptic weight development in cat in our previous work (Bhaumik and Mathur, 2003; Siddiqui and Bhaumik, 2011) the diffusive cooperation between neighboring cells models lateral spread of activation (Bi and Poo, 2001) both at presynaptic level (Bonhoeffer et al., 1989; Schuman and Madison, 1994; Tao et al., 2001a) and at post-synaptic level (Bradler and Barrioneuvo, 1989; Muller et al., 1995; Engert and Bonhoeffer, 1997). In pre-synaptic spread of activation potentiation at synapses spreads to other synapses formed by the same presynaptic neuron to other post-synaptic neurons in the neighborhood (Bonhoeffer et al., 1989; Schuman and Madison, 1994). The spread of potentiation on different post-synaptic cells was modeled in our earlier papers (Bhaumik and Mathur, 2003; Siddiqui and Bhaumik, 2011) through the cortical diffusion term. The post-synaptic spread of potentiation occurs from same post-synaptic cell to different presynaptic cell axons within approximately 70 μm from the site of potentiation (Engert and Bonhoeffer, 1997). The post-synaptic lateral spread of potentiation is modeled in the present as well as in our earlier papers (Bhaumik and Mathur, 2003; Siddiqui and Bhaumik, 2011) through the LGN diffusion term.

Unlike in our earlier model for the synaptic weight development in cats (Bhaumik and Mathur, 2003; Siddiqui and Bhaumik, 2011), we do not invoke diffusive cooperation between neighboring post-synaptic cells for synaptic weight development in mouse. For thalamo-cortical synaptic weight development in mouse we have incorporated only post-synaptic spread of potentiation. Presence of post-synaptic but no presynaptic spread is reported in retinotectal system in the developing Xenopus (Bi and Poo, 2001; Tao et al., 2001b). In retinotectal system in the developing Xenopus potentiation in one RGC pathway spreads to other retinal inputs on the same tectal neuron. But synapses on other adjacent tectal neurons are not affected (Bi and Poo, 2001; Tao et al., 2001b). Such spread of post-synaptic potentiation is attributed to signaling within the post-synaptic cytoplasm (Bi and Poo, 2001; Tao et al., 2001b). Absence of diffusive cooperation between cortical cells results in salt-and-pepper organization in OR preference map and is discussed in the Result section.

### Thalamo-cortical synaptic weight development

In our model *W*^*l*^_*IJ*_ corresponds to the strength of the connection from left eye specific LGN cell at location J to the cortical cell at location I. It is understood in our model that positive values of *W*^*l*^_*IJ*_ represent connection strength from the ON center LGN cell, while negative values represent the strength from OFF center. A similar interpretation is given to *W*^*r*^_*IJ*_ which denote strengths corresponding to the right eye specific LGN cells.

(1) $$\begin{array}{l} \frac{\partial W_{IJ}^{l}}{\partial t}=\left(\gamma_{1}^{l}-K_{1J}^{l}\right)\left(\gamma_{2}-K_{2I}^{l}\right)A_{R}(I,J)A_{J}^{l}W_{IJ}^{l}\\ \;+\;D_{L}(t)\nabla_{J}^{2}W_{IJ}^{l} \end{array}$$

A similar equation holds for *W*^*r*^_*IJ*_. The notation is explained below.

γ^*l*^_1_ = total presynaptic resources available at left eye specific LGN cell at location J. The available resource is assumed to be independent of J.

$$\begin{array}{l} K_{1J}^{l}=\theta \left(W_{IJ}^{l}\right)\left(\sqrt{\sum_{P}{\left(W_{PJ}^{l}\theta (W_{PJ}^{l})\right)}^{2}}\right)\\ \;+\;\theta (-W_{IJ}^{l})\left(\sqrt{\sum_{P}{\left(W_{PJ}^{l}\theta (-W_{PJ}^{l})\right)}^{2}}\right) \end{array}$$

is the total pre-synaptic resource consumed at J. Here θ (*x*) is a step function equal to 1 for *x* > 0 and 0 for *x* < 0. Thus, *K*^*l*^_1*J*_ gets contribution only from connections to cortical cells *P* if *W*^*l*^_*IJ*_ is positive (that is LGN cell is ON type) or other cortical cells *P* connected to OFF LGN cell at J if *W*^*l*^_*IJ*_ is negative. The range of summation for *P* is over the two-dimensional array of *N* × *N* cortical layer.

Mechanisms behind competition between different branches of a single axon are reviewed in Kalil et al. (2011). It is suggested that long distance signaling events mediate the transport of rate-limiting resources such as tubulin to the winning branch and thereby deprive other branches of the opportunity to grow (Butz et al., 2009; Van Ooyen, 2011). The factor (γ^*l*^_1_ − *K*^*l*^_1*J*_) enforces competition for resources among axonal branches in a left eye specific LGN cell.

γ_2_ = total post-synaptic resources available at cortical cell at location I. Cortical resource available is assumed to be independent of I.

*K*^*l*^_2*I*_ = total post-synaptic resources consumed by cortical cell at location I. For connection update in monocular zone in the cortex $K_{2I}^{l}=\left({\sqrt{\sum_{Q}\left(W_{IQ}^{l}\right)}}^{2}\right)$.

For binocular zone, $K_{2I}^{l}=\left(\sqrt{\sum_{Q}{\left(W_{IQ}^{l}\right)}^{2}+{\left(W_{IQ}^{r}\right)}^{2}}\right)$. Here sum over Q is over all the *M* × *M* cells in the LGN layers. Those with positive values of *W*^*l*^_*IQ*_ correspond to ON and those with negative values correspond to OFF layer.

A post-synaptic cortical cell supports a limited number of pre-synaptic left and right eye specific LGN cell connections depending on its post-synaptic resource. The factor (γ_2_ − *K*^*l*^_2*I*_) enforces competition among LGN cells for target space in the cortex.

The arbor function, *A*_*R*_(*I, J*) defines the region from where a cortical cell receives its initial unorganized thalamic afferents (Miller, 1994). The amount of afferents a cell receives, is determined by the arbor window. A square window has been used for the results reported here. It has been shown earlier (Bhaumik and Mathur, 2003) that RF structure does not depend on the type of window used, be it trapezoidal or square.

*A*^*l*^_*J*_ is the activity of the left eye specific LGN cell in location “J.” While updating a synaptic weight between a cortical cell and an LGN cell, the particular LGN cell has to be active, i.e., *A*^*l*^_*J*_ = 1. If an LGN cell is inactive during weight update then the corresponding synaptic weight may decay unless helped by neighboring same-type cells. Before the eye opening in mouse, a dense activation pattern of neurons in the visual cortex is reported (Rochefort et al., 2009). While updating *W*^*l*^_*IJ*_ we assume that cortical cell at location I is active.

In Equation (1), the last term models post-synaptic lateral spread of potentiation (Bradler and Barrioneuvo, 1989; Muller et al., 1995; Engert and Bonhoeffer, 1997) on neighboring LGN synapses on the same post-synaptic neuron. Engert and Bonhoeffer (1997) through two sets of elegant experiments, have shown that long-term potentiation (LTP) can spread along the post-synaptic dendrite from activated synapses to nearby synapses regardless of the history of activation of these synapses. The spread of LTP occurs to nearby synapses located within 70 μm distance of activated synapse. D_L_(t) is the LGN diffusion constant and is given below.

$${\text{D}}_{\text{L}}\left(\text{t}\right)=\{\begin{array}{c} {\text{D}}_{\text{L}},\text{ for t}\leq {\text{t}}_{\text{c}}\\ \text{0},\text{ for t}>{\text{t}}_{\text{c}} \end{array}$$

where, t_c_ is the closing time for post-synaptic lateral spread of potentiation in LGN. For t > t_c_ the synaptic weight modification is only governed by the reaction term. The subregion structure in the RF does not change—only the magnitude of weights change till the resources last and a steady state is reached. At 2000 epoch the subfields in the RF are well-formed and the RF structure does not depend on type of window used for D_L_ (t) whether it progressively declines from D_L_ to zero or abruptly becomes zero at t = t_c_.

The model parameter values in the reaction diffusion equation determine (i) the number of sub regions and the structure of the receptive filed (RF) of cortical cells and (ii) the 2D organization of RFs in the model cortex. The structure of RF determines the cortical cell's orientation tuning and spatial frequency selectivity. The 2D organization of RFs determine orientation map. The effects of varying the model parameters, the value of D_L_, the LGN resource γ^*l*^_1_ and the cortical resource γ_2_ are given in detail in Bhaumik and Mathur (2003). The number of sub fields in the RF of a cortical cell increases as D_L_ is reduced (see **Figure 9** in Bhaumik and Mathur, 2003). The value of LGN resource γ^*l*^_1_ does not affect the structure of RF and the number of subregions (see **Figure 6** in Bhaumik and Mathur, 2003). For low values of LGN resource γ^*l*^_1_, synaptic strengths are quite weak due to scarcity of resources and as a result the cell is not fully responsive to input stimuli. As the resources are increased, the synaptic weights become stronger, without affecting the number of sub regions and the structure of the RF. LGN cells compete for cortical resource γ_2_. For low values γ_2_, the number of simple cells with one subregion is high. The synaptic strengths for cells with two or three subregions are too weak for γ_2_ ≤ 0.5. With increase in γ_2_, the number of cells with two and three subfields increases. For γ_2_ ≥ 1, with increase in cortical resources, the synaptic strengths increase, but the number of subfields remains the same (see **Figure 5** in Bhaumik and Mathur, 2003).

We introduce a subregion correspondence factor, *C*^*l*^, in Equation (1) for synaptic weight development for cortical cells in binocular zone during the critical period when subregions or subfields correspondence develops. During the critical period the evolution of connection strength *W*^*l*^_*IJ*_ is governed by the equation

(2) $$\begin{array}{l} \frac{\partial W_{IJ}^{l}}{\partial t}=\left(\gamma_{1}^{l}-K_{1J}^{l}\right)\left(\gamma_{2}-K_{2I}^{l}\right)A_{R}(I,J)A_{J}^{l}C^{l}W_{IJ}^{l}\\ \;+D_{L}(t)\nabla_{J}^{2}W_{IJ}^{l} \end{array}$$

A similar equation is used for *W*^*r*^_*IJ*_.

For updating *W*^*l*^_*IJ*_, the subfield correspondence factor *C*^*l*^ (Siddiqui and Bhaumik, 2011) is defined as

(3) $$C^{l}=\{\begin{array}{l} +\text{1 if }W_{IJ}^{l}\text{ and }W_{IJ}^{r}\text{ are of the same sign}\\ \begin{array}{cc} -1 & \text{ otherwise} \end{array} \end{array}$$

A plausible biological basis for C^l^ is the N-methyl-D-asparate receptors (NMDARs)-mediated synaptic potentiation. A presynaptic spike causes the release of glutamate, which binds to the NMDARs. NMDA channels allow influx of Ca^++^ (Karmarkar and Buonomano, 2002). Larger calcium influx could produce synaptic potentiation (Lisman, 1989) whereas smaller calcium influx could produce depression (Yuste et al., 1999). In our model, C^l^ = +1, when at LGN location “J” synaptic connections from both the left and the right eyes are either ON type or OFF type. The active presynaptic inputs from the left and the right eye specific LGN cells at “J,” add at the post-synaptic cell and W^l^_IJ_ grows. For C^l^ = −1, synaptic connection from the left eye is ON (OFF) type but synaptic connection from the right eye is OFF (ON) type. Thus, both the pre-synaptic inputs are not active at the same time and W^l^_IJ_ decays unless it is supported sufficiently by the neighbors through the diffusive term. Had we not included C^l^ in Equation (2), irrespective of whether the synaptic connections from the left and the right eyes from LGN location “J” are both same (ON-ON or OFF-OFF) type or not, W^l^_IJ_ grows and the left and the right eyes RFs of a cortical cell do not develop subregions or subfields correspondence i.e., the RFs in the two eyes develop independently.

Synaptic plasticity in cortex is age dependent (Kirkwood et al., 1997; de Marchena et al., 2008). Age dependent decline in visual cortical plasticity may be due to changes in the NMDA receptor gating properties (Carmignoto and Vicini, 1992). Further the mechanisms of synaptic plasticity differ across cortical layers. A laminar progression of plasticity is reported in mouse visual cortex with critical period in layer IV lasting for a short period as compared to layer II/III (Jiang et al., 2007).

The duration of NMDA-mediated excitatory post-synaptic currents (EPSCs) last longer in young animals as compared to adults. A longer lasting NMDA mediated EPSC serves to strengthening of synaptic connections (Carmignoto and Vicini, 1992). It is suggested that composition of NMDARs at young synapses allows potentiation to occur at lower threshold (de Marchena et al., 2008). What factors determine the threshold for modifying synapses and how the magnitude of synaptic modifications are regulated in early period of development in visual cortex, particularly before eye opening are mostly unknown.

## Results

Each cortical neuron in monocular zone receives synaptic connections from the left eye specific LGN layers. Each cortical neuron in binocular zone receives synaptic connections from both the left and the right eye specific LGN layers. Synaptic connections are developed using reaction-diffusion model equations with circular boundary condition. The initial synaptic weights are picked from uniform random distribution of weights of the order of 10^−6^. For the results presented in this paper the t_c_, the closing time for post-synaptic lateral spread of potentiation in LGN is 3000 epochs. LGN diffusion constant value D_L_ = 0.0055 for t ≤ t_c_. The cortical resource, γ_2_ = 1.5. Contra-lateral eye makes stronger connections as compared to ipsi-lateral eye (Coleman et al., 2009). Thus, in our model LGN resources of contra-lateral eye (Left eye here), γ^*l*^_1_ = 1.2 and that of ipsi-lateral eye (Right eye here), γ^*r*^_1_ = 0.8.

In the monocular zone, synaptic connections for cortical cells are developed using reaction-diffusion equation given in Equation (1). In absence of diffusive spread of potentiation on neighboring LGN synapses on the same post-synaptic neuron i.e., with the LGN diffusion constant D_L_ = 0, the synaptic weight development is governed by only the reaction term and developed RFs have random distribution of ON and OFF synaptic weights. No ON or OFF sub field develops in absence of LGN diffusion. When both reaction and diffusion term are included for development of synaptic weights as in Equation (1), from the initial random distribution of synaptic weights at *t* = 0, the relatively higher values of synaptic weights acts as nucleation centers and at around epoch 100, the left and the right RFs of the cortical cell develop small patches of ON or OFF subregions. The formations of patches occur due to cooperation among ON (OFF) synapses helping other neighboring ON (OFF) synapses to grow and push out any OFF (ON) synapses existing in a patch. The cooperation phenomenon is gradual and is due to diffusion in the LGN. At epoch 3000, RFs have well-defined segregated ON and OFF subregions with gradual transition from ON (OFF) subregion to OFF (ON) subregions. At t = t_c_ = 3000 epochs, the utilization of resources are almost saturated, and the change in synaptic weights are negligible. For t>3000 epochs the synaptic weight modification is only governed by the reaction term. The subregion structure in the RF does not change—only the magnitude of weights change till the resources last and a steady state is reached.

To achieve computational speed, we assume LGN cell and its neighbors are active during weight update for the results presented in this article. In the Supplementary Material we have shown that the nature of RFs developed with spontaneous activity in LGN remains qualitatively same.

**Figure 3:**
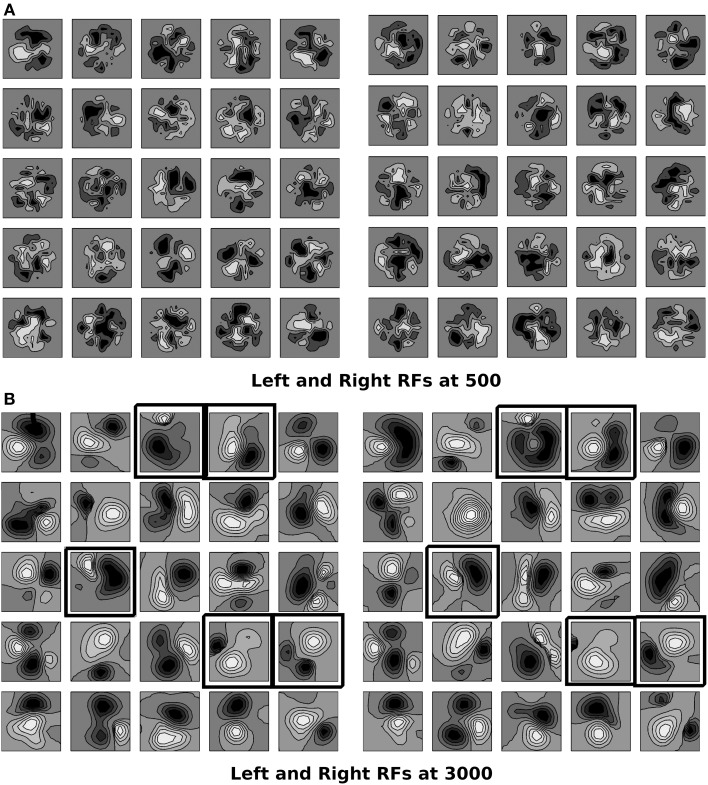
**(A,B)** The left and the right RFs for a 5 × 5 section of cortex in the binocular zone are shown at 500 epochs, and 3000 epoch. RFs are locally diverse where near-by neurons have largely dissimilar receptive fields. The ON and OFF subregions are shown in Gray-scale with white (black) color representing connections from ON (OFF) LGN cells. The shading is proportional to strength of connection from LGN cells. At 500, epochs the sub-field structure is found only in few cells. The left and the right RFs are not similar as sub region correspondence factor is not included before 500th epoch. At 3000, the sub-field structure is visible. Due to sub region correspondence factor acting between 500 and 3000 epochs, the left and the right RFs become similar. Black boxes are marked around RFs for cells which are oriented in left and right eyes individually and the orientation preference difference between the left and the right eyes is less than 30°.

Critical period is the period of heightened plasticity during which the orientation alignment between the left and the right eye receptive fields for cortical cells in the binocular zone takes place in normal development. Wang et al. (2010) reported that in mice, the left and the right eye receptive fields have poor matching of preferred orientation at the start of the critical period but they become matched by the end of it. Let t = *C*_*iter*_ be the epoch at which the critical period starts and t = t_c_ be the epoch for the end of critical period for orientation plasticity. In binocular zone therefore, we allow the left and the right eye receptive fields to develop independently till 500 epochs using Equation (1). From 500 to 3000 epochs we develop synaptic weights for binocular cells using Equation (2) wherein subregion correspondence factor is included in the reaction diffusion equation. The left and the right eye RFs at 500 and 3000 epochs for a 5 × 5 section of cortex in the binocular zone are shown in Figure 3. According to the feed-forward model proposed by Hubel and Wiesel (1962), orientation selectivity arises from specific arrangement of geniculate inputs. Ours is a feed forward model and the preferred orientation of modeled cortical cells is determined by the layout of the elongated ON and OFF subregions as shown in Figure 3B. RFs are locally highly diverse, with nearby neurons having largely dissimilar receptive fields. The ON and the OFF subregions are shown in Gray-scale with white (black) color representing strong synaptic connection from ON (OFF) LGN cells. The shading is proportional to the strength of the ON/OFF synaptic connections from the LGN cells. The choice of 500–3000 epochs as time window for critical period for orientation plasticity is justified later in this section.

### Cell response

To obtain cell response using SRM moving sinusoidal gratings at 90% contrast (Grubb and Thompson, 2003) were used. The velocity of gratings was fixed at 100°/s orthogonal to the orientation. This results in temporal frequency of 4Hz for sinusoidal grating with spatial frequency of 0.04 cycles/°. Stimulus orientation was varied from 0° to 180° in steps of 18°. Peri-Stimulus time histogram (PSTH) was made for each response by binning the response in bins of 100 ms each and calculating the spike rate in each of these bins. Spike rates per second were computed for individual bins and the response was then averaged over the 30-recorded Peri-Stimulus time histograms. The cell spike response for any given orientation of input stimulus is the maximum response obtained in the averaged histogram. The mean spike rate for binocular cells is 22 spikes/s and for left monocular cells it is 19 spikes/s. This agrees with data in literature (Metin et al., 1988).

Approximately 60% non-orientation selective cells are reported in mouse V1 (Mangini and Pearlman, 1980; Metin et al., 1988; Hübener, 2003) i.e., around 40% cells are orientation selective. In our study, in the binocular zone we observed 39% orientation selective cells when stimulated binocularly. In monocular zone 38% cells are orientation selective.

**Figure 4:**
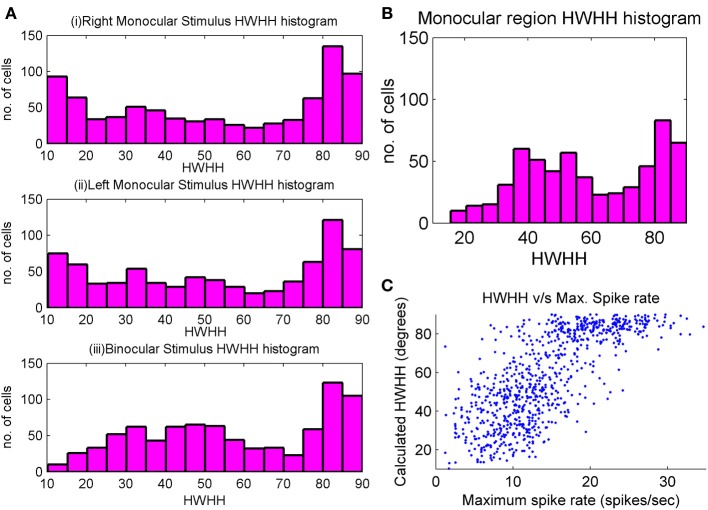
**(A)** HWHH histogram of the left monocular, the right monocular, and the binocular responses for cortical cells in a 35 × 60 section inside the binocular region. There is a large chunk of cells which are poorly orientation tuned, but there is also a large number of cells with good orientation tuning. Tuning properties of monocular responses are better than binocular responses. **(B)** HWHH histogram for a 35 × 60 section of cells in the monocular region. **(C)** Scatter plot between HWHH and maximum spike rate (spikes/s) of binocular response for a 35 × 60 section of cells in the binocular region of the cortex.

Some studies (Metin et al., 1988; Niell and Stryker, 2008) report that the tuning width of mouse cells is very good, around 20° while others (Metin et al., 1988; Van Hooser, 2007) report that they could not get many highly tuned cells when inhibition was turned off. In our study we have feedforward excitatory connections from LGN to cortex. We get some cells that are highly tuned, while there is also a considerable number that have large HWHH and hence, are poorly tuned. Cells with three subregions shows better tuning as compared to cells with two subregions, particularly if one of the two subregion is bigger in size. Also cells with patchy subregions i.e., not well-formed subregions are poorly orientation selective. For cortical cells in binocular region, HWHH histogram for right monocular, left monocular, and binocular responses are shown in Figure 4A. A number of cells have low values of HWHH (≤20°) in their monocular response. However, only those cells that do not have large difference between the left and the right eye orientation preference have low values of HWHH in binocular response. HWHH histogram for cortical cells in monocular region is shown in Figure 4B. Note that the HWHH histogram for cells in monocular region is similar to the HWHH histogram for binocular response of cells in binocular region. This is because spike rates for monocular cells are similar to the spike rates for binocular response in binocular cells. Figure 4C depicts spike rate as function of HWHH for cells in binocular zone. We observe that for low spike rate, we are more likely to get better orientation tuning.

We have also obtained OSI of the modeled cells. OSI is calculated by making a vector for each orientation response with magnitude equal to the magnitude of response and angle being twice the stimulus orientation. The OSI is calculated using the relation (Tan et al., 2011)

$$OSI=\frac{\sqrt{{\left(\sum_{\theta }R_{\theta }sin(2\theta )\right)}^{2}+{\left(\sum_{\theta }R_{\theta }cos(2\theta )\right)}^{2}}}{\sum_{\theta }R_{\theta }}$$

where R_θ_ is the response for stimulus orientation angle θ. OSI value varies between 0 and 1. Median OSI value in mouse is 0.31 (Tan et al., 2011). In the model cortex there is a large chunk of cells, which are poorly orientation tuned (*OSI* < 0.3), but there is also a large number of cells that show good orientation tuning (*OSI* > 0.3). This result is nearer to that observed in Hagihara and Ohki (2013).

**Figure 5:**
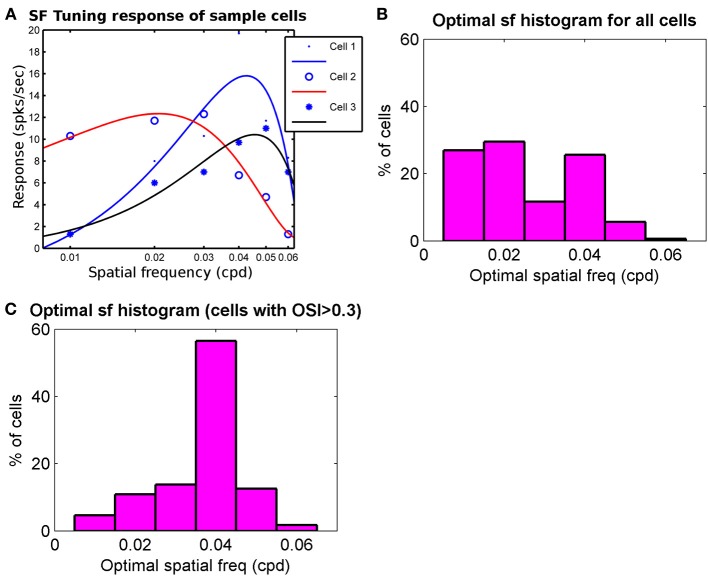
**(A)** The spatial frequency response of 3 sample cells. The response at sample frequencies (0.01–0.06 cycles/° with intervals of 0.01 cycles/°) and velocity of 100°/s is fitted with cubic spline to determine optimal spatial frequency. Optimal spatial frequency for Cell 1–Cell 3 are 0.042 cycles/°, 0.022 cycles/°, and 0.048 cycles/°, respectively. **(B)** The Optimal spatial frequency histogram for all cells (*N* = 374) from a patch in binocular region that are oriented in left and right RFs and have ΔOR < 30°. **(C)** Optimal spatial frequency histogram for cells from **(B)** that also have moderate orientation tuning (OSI > 0.3). Mean in **(C)** is 0.038 cycles/°.

Reported optimal spatial frequency range across the cell population in mice ranges from 0.02–0.09 cycles/° (Gao et al., 2010) to 0.003–0.1 cycles/° (Van den Bergh et al., 2010; Vreysen et al., 2012). We have studied the spatial frequency response of our modeled cells. For obtaining spatial frequency response a centrally located cortical section in binocular region was chosen. Further, cells (*N* = 374) that are orientation selective in their binocular response and have difference in the preferred orientation, ΔOR < 30°, between left eye and right response were selected. The spatial frequency responses of these cells were obtained by exposing the cells to drifting gratings at preferred orientation as obtained from their binocular response, at different spatial frequencies but at same velocity (100°/s). The spatial frequency responses of three sample cells are shown in Figure 5A. The histogram for the optimal spatial frequency is shown in Figure 5B. For cells (*N* = 138) with at least moderate tuning (*OSI* > 0.3) the spatial frequency histogram is shown in Figure 5C. The mean preferred spatial frequency of these cells is 0.038 cycles/° and close to the values reported (Gao et al., 2010; Van den Bergh et al., 2010; Vreysen et al., 2012).

### OR preference and OD map

**Figure 6:**
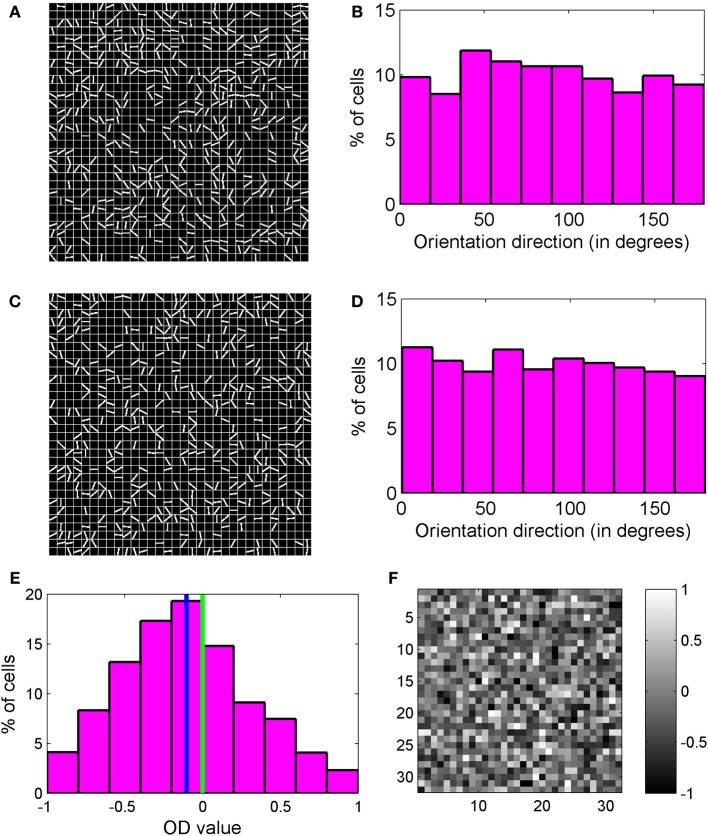
**(A,C)** Two orientation preference maps for a 32 × 32 section of cortex inside binocular region and monocular region, respectively. The lines depict orientation preference for cells. For cells that are not orientation tuned, no line is shown. We observe a salt and pepper orientation preference map. **(B)** Orientation preference histogram for binocular cells. **(D)** Orientation preference histogram for monocular cells. All orientations are almost equally present. **(E)** OD histogram of the cells in binocular region. Mean OD is −0.1031 and depicts contra-lateral dominance. **(F)** OD map for the section of the cortex shown in **(A)**. The OD map is unstructured.

Two orientation preference maps for 32 × 32 section of cortex inside binocular region and monocular region are shown in Figures 6A,C. The white color lines depict the binocular preferred OR of cells. No orientation line is shown for the cells that are not orientation tuned. The orientation preferences of nearby cortical cells are uncorrelated and we observe a salt and pepper OR preference map. In cat visual cortex, neurons are arranged according to their preferred orientation resulting in smooth OR preference map with pinwheels. In mice there is no evidence of such smooth OR preference map (Dräger, 1975; Mangini and Pearlman, 1980; Metin et al., 1988; Bonin et al., 2011) although neurons are tuned to orientation of the visual stimulus. In a salt-and-pepper organization of OR preference map, the receptive fields with different preferred orientations could sample a visual scene uniformly. In cats and ferrets with structured OR preference maps at certain visual field positions some degree of overrepresentation of certain orientations are likely. But in combination with eye movements this disadvantage may be overcome (Kaschube, 2014).

Hubel and Wiesel (1963) had suggested that smooth OR preference map in cat visual cortex may lead to wiring length minimization in cortex. More recently it is proposed that the differences in the structure of orientation maps in different species may be explained by differences in intra-cortical circuits and wiring length minimization (Koulakov and Chklovskii, 2001). One of us has shown (Bhaumik and Mathur, 2003; Siddiqui and Bhaumik, 2011) that local diffusive interaction among cortical cells results in formation of smooth OR preference map with pinwheels. Lack of diffusive cooperation among neighboring cortical cells results in salt and pepper organization of OR preference maps as shown in Figures 6A,C. We, therefore, suggest that a lack of columnar organization in OR in rodents is due to absence of diffusive cooperation among neighboring cortical cells in these animals during development.

Formation of RFs with ON/OFF subregions in orientation selective visual cortical cells is determined by local diffusive interaction among neighboring LGN cells. Hence development of orientation selectivity takes place irrespective of presence or absence of columnar organization of orientation selectivity. The reaction diffusion model used by us in this paper for mouse and in our earlier work on cat (Bhaumik and Mathur, 2003; Siddiqui and Bhaumik, 2011) can be used to study the formation of RF of orientation selective cells in diverse animals such as mice or rats with no OR preference map to ferrets and cats with smooth OR preference map with pinwheels. Orientation preference histogram for binocular cells and monocular cells are shown in Figures 6B,D. All orientations are almost equally present. 39% of cells in binocular zone in our model cortex are orientation selective.

OD for cells in binocular region is a measure of dominance of response of one eye over the other. OD value for our modeled cells is calculated as:

$$\text{OD}=\frac{\text{R}-\text{L}}{\text{R}+\text{L}}\text{ where},\text{ L}=\sum_{\theta }{\text{R}}_{\text{l}}(\theta )\text{ and R}=\sum_{\theta }{\text{R}}_{\text{r}}(\theta )$$

R_1_(θ) is the response when stimulus is applied to only the left eye and R_r_(θ) is the response to stimulus applied only to the right eye. θ is stimulus orientation angle. OD histogram for cells in binocular zone is shown in Figure 6E. OD lies between −1 and +1. Negative OD values correspond to left eye dominance and positive OD values correspond to right eye dominance. OD values near 0 indicate binocularity. The cell population shows contra-lateral dominance with mean OD of −0.1031. The OD map for the same 32 × 32 section of cortex considered earlier for Figure 6A, is shown in Figure 6F. The OD map is unstructured.

### Critical period and orientation plasticity

In mouse, the critical period starts at around P21 and lasts till P35 (Huberman et al., 2008). Mice aged between P20–P23 and P31–P36 are used to study binocular orientation preference matching (Wang et al., 2010). In our study 500 epoch and 3000 epoch corresponds to start and end of orientation plasticity. There is abundance of literature on ocular dominance plasticity (ODP). Mechanisms such as long-term depression (LTD), LTP and neurotropic signaling are proposed for ODP (Espinosa and Stryker, 2012). Literature on Orientation plasticity is limited (Wang et al., 2010; Sarnaik et al., 2014) and no mechanism has been proposed.

**Figure 7:**
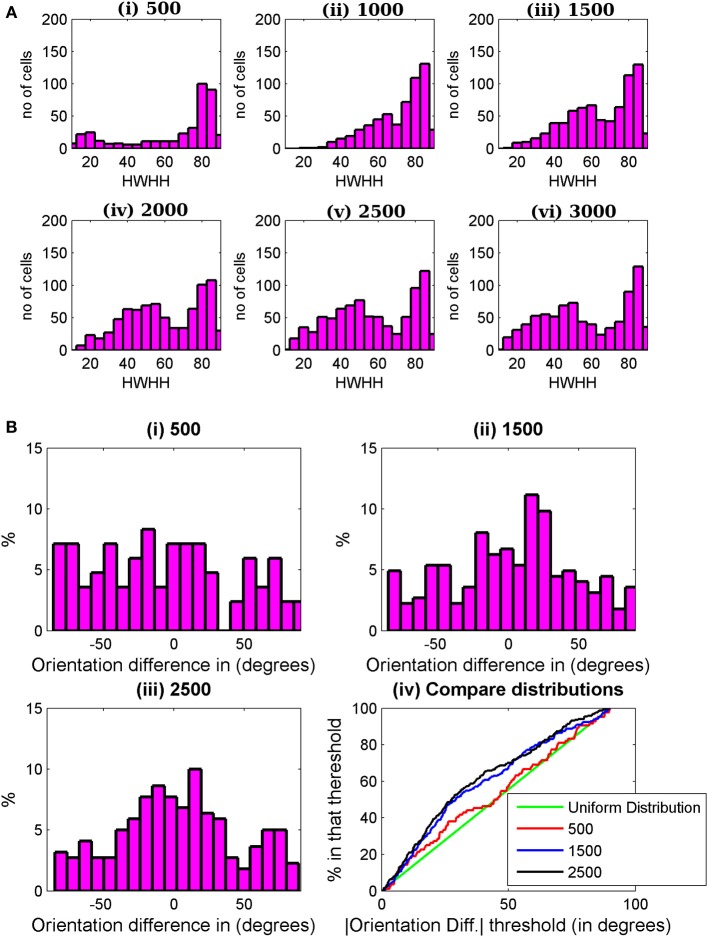
**(A)** HWHH histogram for the cells in a 35 × 60 patch from the binocular region in the cortex. The histograms are shown from 500 to 3000 epochs at an interval of 500 epoch. **(B)** Histograms of preferred orientation difference between the two eyes at **(i)** 500, **(ii)** 1500, and **(iii)** 2500 epochs. In **(iv)** the histograms in **(i)**–**(iii)** are compared with uniform distribution (green line). Uniform distribution is the expected distribution of |ΔOR| when the left and right receptive fields develop independently. At 500 epochs, |ΔOR| distribution is almost uniform. There is not much improvement in |ΔOR| between 1500 and 2500 epochs.

For binocular RF development prior to the critical period, we use Equation (1) till 500 epochs. We start the iteration with very small (of the order of 10^−6^) random values of synaptic weights. Due to reaction term, the weights quickly increase and observable responses from cells with orientation tuning start from 500 epochs onward. A significant number (approximately 20%) cells display orientation tuned monocular responses at 500 epochs. But as the receptive field in two eyes develop independently, only 4% cells have both tuned left and right monocular responses with absolute value of |OD| < 0.8 and maximum spike rate in left and right monocular responses greater than 3 spikes/s (baseline firing rate of cortical cells). Experimentally oriented cells are observed at the start of critical period (Wang et al., 2010). In our simulation we take 500 epochs as the start of the critical period. We develop synaptic weight using Equation (2) from 500 to 3000 epochs. Histogram of HWHH of binocular cells is shown in Figure 7A starting from 500 epochs to 3000 epochs at an interval of 500 epochs. With increase in epochs the number of binocular orientated cells increases from 4% at 500 epochs to 39% at 3000 epochs. The increase in number of cells with better binocular tuning is due to the improvement of orientation tuning in monocular responses as well as improved alignment of orientation preferences between the two eyes.

During the critical period the alignment of the left and the right eye preferred orientation takes place. Figures 7Bi–iii depicts the histograms of preferred orientation difference between the two eyes at 500, 1500, and 2500 epochs. With increasing number of epochs, the number of binocular orientated cells increases along with improvement in alignment between preferred orientations of the two eyes. Figure 7Biv shows a plot of cumulative distribution of the percentage of cells as a function of preferred orientation difference between the two eyes, ΔOR, from 500 epochs to 2500 epochs at an interval of 1000 epochs. The green line in the plot indicates uniformly distributed orientation difference. Note that at 500 epochs, the orientation difference distribution is almost uniform. This is expected because till 500 epochs the RFs of the two eyes developed independently. The preferred orientation alignment improves significantly up to 1500 epochs; thereafter, it is slower. We take 3000 epochs as the closing of the time window for orientation plasticity in juvenile mouse. How the choice of starting and closing time for the time window for orientation plasticity affects the preferred orientation alignment between the two eyes and the binocular orientation tuning is discussed next.

### Start and end of critical period

The choice for deciding on the starting time (C-iter) for the critical period for orientation plasticity is guided by the following three factors.

1. At the beginning of the critical period there must be some cells that are orientation tuned in both eyes so that the preferred orientation for the two eyes can be ascertained. Also, the preferred orientation in most of the cortical cells should be mismatched between the two eyes.
2. Similarity of orientation preference between the two eyes should improve during the critical period. At the end of critical period (3000 epoch in our case) we must have similar amount of alignment of preferred orientation of the two eyes as reported in juvenile mice.
3. At the end of plasticity time window approximately 40% cells should be orientation tuned as reported in literature.

**Figure 8:**
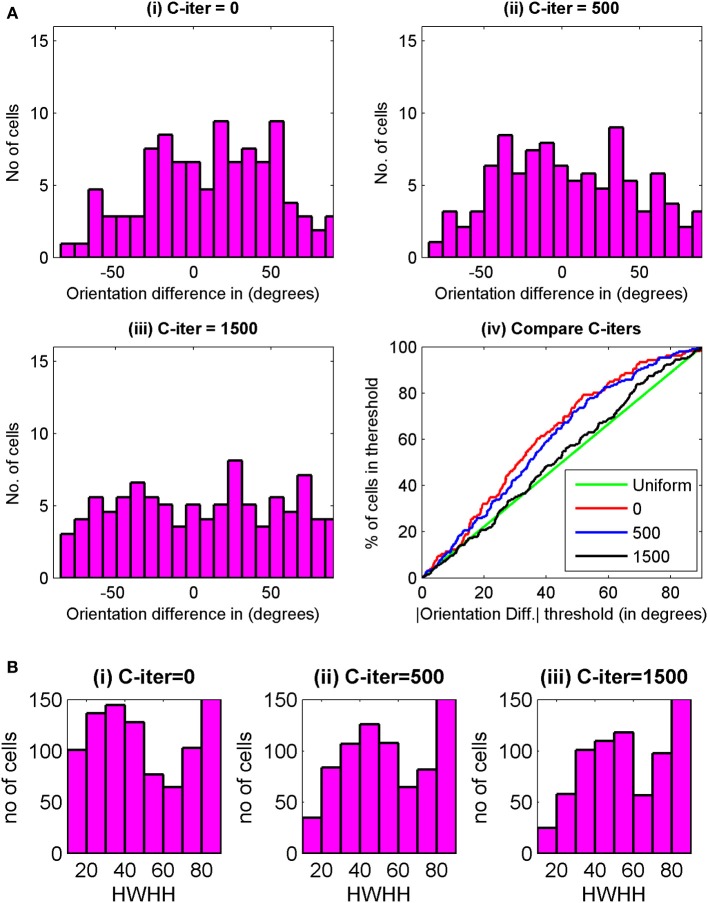
**(Ai–iii)** Histogram of preferred orientation difference between the two eyes, ΔOR, at maturity (3000th epochs) for C-iter = 0, 500, and 1500 for cells from the same 35 × 60 patch of cortex used for Figure 7. **(iv)** The cumulative distribution of ΔOR, at 3000 epochs is shown for three different values of C-iter. The green line depicts uniform distribution. **(B)** The HWHH histograms for binocular oriented cells from the same patch of cells as in **(A)** for **(i)** C-iter = 0, **(ii)** C-iter = 500, and **(iii)** C-iter = 1500.

Histogram of preferred orientation between two eyes, ΔOR, at 3000 epochs for C-iter = 0, 500, and 1500 epochs are shown in Figures 8Ai–iii. We have plotted in Figure 8Aiv the cumulative distribution of ΔOR, at 3000 epochs for three different C-iter values. The green line depicts uniform distribution. Best orientation alignment for the two eyes are obtained for C-iter = 0 epoch. However, at 0th epoch no cells are orientation tuned, thus first condition for choice of C-iter is not satisfied. For C-iter = 1500 epochs the alignment in preferred orientation is poor and the cumulative distribution is almost similar to random distribution. With C-iter = 500 epochs, at 3000 epoch we get 39% orientation selective cells in the binocular zone with orientation alignment similar to the ones reported in literature (Wang et al., 2010). The HWHH of cells for binocular input at maturity for three different C-iter values are shown in Figure 8B. The mean HWHHs are 51.45°, 57.27°, and 58.41° at C-iter = 0, 500, and 1500 epochs. Note that the mean HWHH data contains all orientation selective cells i.e., cells with HWHH ≤ 90°. If we consider only moderate to high orientation selective cells i.e., cells with *OSI* > 0.3 the mean HWHH improves. For instance for C-iter = 500, at 3000 epoch mean HWHH is 36° for cells with *OSI* > 0.3.

The end of critical period for orientation plasticity is determined by two factors.

1. Orientation tuning, and
2. Alignment of preferred orientation between the two eyes.

**Figure 9:**
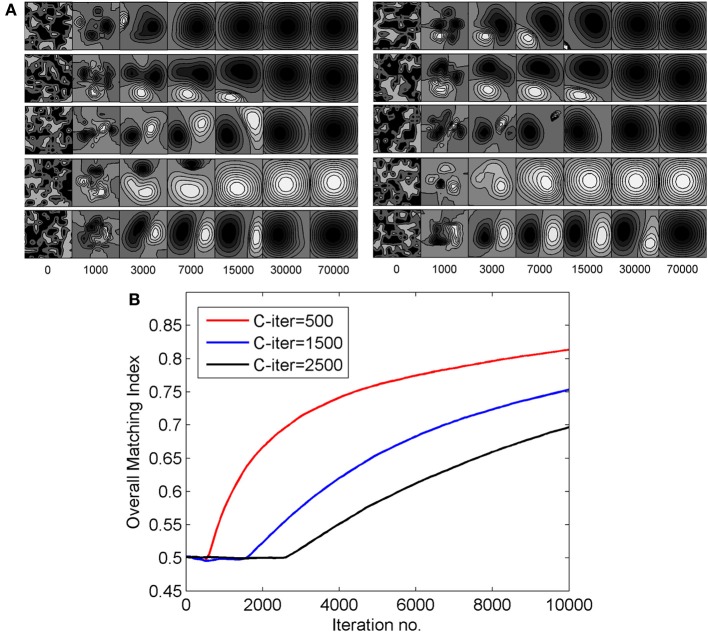
**(A)** The effect of extending the duration of critical period on RFs. The left eye and the right eye specific RFs for some sample cells are shown. The RFs shown on the left of the figure are for the left eye and those shown on the right are for the right eye. **(B)** The change in overall matching index for three different values of C-iter—500, 1500, and 2500 epochs, respectively.

In the result presented in this paper 3000 epochs is taken as the time for end of critical period. To study the effect of extending the duration of critical period on RFs, we carried on synaptic weight development for different values of t_c_, ranging from 3000 epoch to 70,000 epoch. Figure 9A shows left and right eye RF for some sample cells from the model cortex for differenr values of t_c_. For larger values of t_c_, the diffusion continues for longer time and the sub-field structure of the receptive fields is lost. Consequently orientation selectivity is also lost. The critical period of plasticity in layer IV in mouse is shorter than that in layer II/III (Jiang et al., 2007). Our study shows that a critical period beyond 2500–3000 epoch in layer IV causes loss in orientation selectivity.

The alignment of preferred orientation comes from subregion correspondence in the RFs of the left and the right eyes. To quantify subregion correspondence we calculate a matching index for RFs for the left and the right eyes. We define the matching index for a cortical cell as the fraction of the weights in left and right receptive fields that have same sign (either ON-ON or OFF-OFF). At each epoch we obtain the matching index for each cortical cell. Next we determine the overall matching index. Overall matching index at an epoch is the matching index averaged across all cortical cells. Matching index is only a measure of subregion/sub-field correspondence and not of orientation alignment. In Figure 9B, we plot the change in overall matching index when RFs were developed with three different values C-iter = 500, 1500, and 2500 epochs. In all the three cases, the matching index is 0.5 before the critical period starts as the left and the right receptive fields for a cortical cell develop independently. After the critical period starts, the matching index increases, as expected. It takes more iterations for matching index to reach similar value in case of C-iter = 2500 compared to C-iter = 500, but orientation tuning is lost as the number of epoch increases and we observe receptive fields with blob like single region. The rate of increase in matching index is faster when critical period starts earlier.

Our study predicts that the starting and closing time for critical period during normal development affect the orientation preference alignment between the two eyes and orientation tuning in cortical cells. Therefore, animals with similar OR preference map organization but different developmental time scale are expected to have different distribution of ΔOR and percentage of OR tuned cells. This prediction needs experimental verification.

## Discussion

We have used a reaction diffusion model to develop RFs to study orientation selectivity in mouse visual cortex during the critical period when alignment of orientation preference between the two eyes takes place. Development of orientation selectivity in both monocular and binocular regions are modeled for mice in this paper. For cats we had earlier modeled development of orientation selectivity in binocular region (Siddiqui and Bhaumik, 2011, 2013). In the model mouse cortex binocular zone covers 40° binocular visual space and monocular zone covers 40° left monocular visual space. To obtain cortical cell response using the three layer visual pathway model we have (i) implemented a RGC model for mouse, (ii) developed a SRM for cortical cell to capture realistic spike rates and responses in binocular as well as in monocular region of the mouse cortex, (iii) presented thalamo-cortical synaptic weight development in mice, and (iv) studied the matching of binocular orientation preference during the critical period.

**Figure 10:**
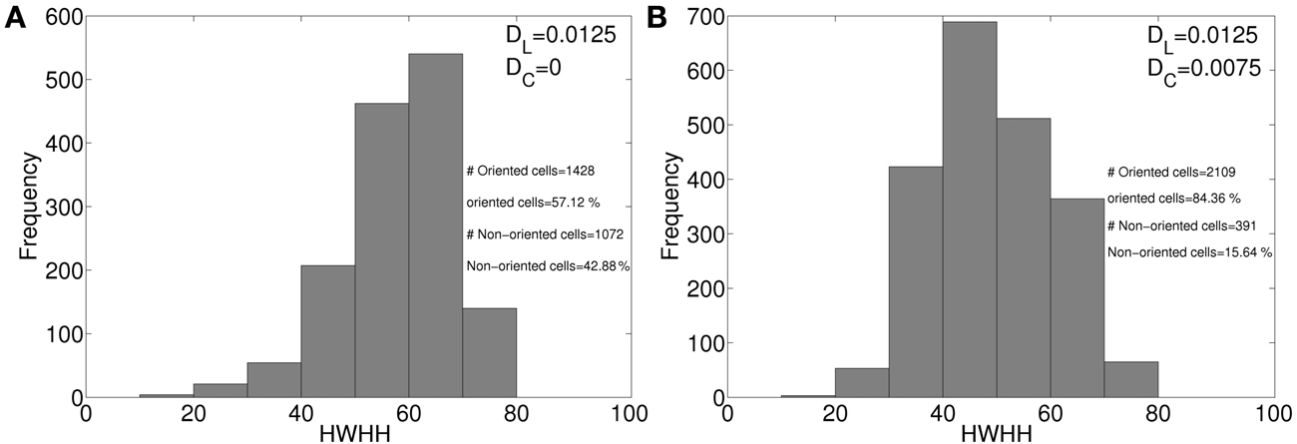
**HWHH histogram in model cat cortex, (A) in absence, and (B) in presence of diffusive cooperation among cortical cells**.

Our model captures the diversity in RFs and orientation preferences (see Figure 3) in local cell population as reported in Bonin et al. (2011). The local diversity in orientation preference in mice is captured in the salt and pepper orientation maps (see Figures 6A,C). Approximately 75% of cells in adult ferret cortex (Chapman and Stryker, 1993) and about 90% of cells in adult cat cortex (Bishop and Henry, 1972) are orientation selective. In mouse V1 around 40% cells are orientation selective (Mangini and Pearlman, 1980; Metin et al., 1988; Hübener, 2003). We attribute the lower % of orientation selective cell in mice as compared to ferrets and cats to absence of diffusive cooperation between neighboring cortical cells during development. Absence of diffusive cooperation between cortical cells also causes salt and pepper orientation map in mice. To get a quantitative understanding of how diffusive cooperation among near neighbor cortical cells during development affects the % of orientation selective cells and tuning properties we studied development of orientation selectivity in our model cat cortex (Siddiqui and Bhaumik, 2011) (i) in absence, and (ii) in presence of diffusive cooperation among cortical cells. The orientation tuning histogram for the two cases are shown in Figures 10A,B, respectively. All parameters for the developing RFs in cat for Figure 10B are same as given in our earlier work (Siddiqui and Bhaumik, 2011). In absence of diffusive cooperation among cortical cells, 57.12% cells are orientation selective as compared to 84.34% when diffusive cooperation is present.

It was suggested that cortical neurons with IDPOs forms “second neural mechanism for depth perception” (Blakemore et al., 1972). Bridge and Cumming (2001) and Bridge et al.'s (2001) study did not support this hypothesis and they showed that V1 cells with IDPO in monkey are not effective in horizontal slant detection. We have recently shown (Siddiqui and Bhaumik, 2013) that cortical cells in V1 do not possess slant selectivity. During normal development binocularly matched orientation preference between the two eyes develops. Cells with IDPO are cells with imperfect binocular matching at the end of the critical period for the alignment of the left and the right eye preferred orientation. We have shown in Table 1 the distribution of cells with IDPO in the model cat visual cortex with and without diffusive cooperation among cortical cells. There is no significant effect of cortical diffusion on orientation preference matching.

**Table 1:** **Distribution of IDPO with and without cortical near neighbor interaction**.

	***DL* = 0.0125 and *DC* = 0.0075**	***DL* = 0.0125 and *DC* = 0**
**Range of |IDPO|**	**% of OR selective cells**	**% of OR selective cells**
0° ≤ |IDPO| <10°	39.165	33.75
10° ≤ |IDPO| <20°	17.73	16.246
20° ≤ |IDPO| <30°	10.905	8.19

We have modeled development of orientation selectivity through connections from thalamus to cortical simple cells in layer IV in mice V1 in the present paper as well as in our earlier work for layer IV in cat V1 (Bhaumik and Mathur, 2003; Siddiqui and Bhaumik, 2011). Orientation selectivity emerges from specific connections from thalamus to cortical simple cells in layer IV (Hubel and Wiesel, 1962; Reid and Alonso, 1995, 1996; Ferrester et al., 1996; Kara et al., 2002; Ohki and Reid, 2007). Orientation selectivity proceeds, or emerges before the clustering of horizontal projections (Sur and Leamey, 2001). At P23 approximately one quarter of ferret cortical neurons studied by Chapman and Stryker (1993) displayed orientation selectivity. Significant Clustering of horizontal connections were seen around P27 (Ruthazer and Stryker, 1996). Recently it is reported that in mice emergence of orientation selectivity does not depend on the precise arrangement of local horizontal connections (Ko et al., 2013). Neurons first acquire feature preference from feedforward inputs before the onset of sensory experience (Ko et al., 2013). At the eye opening neurons are already highly selective for visual stimulus. After eye opening, local connectivity reorganizes extensively. The nascent orientation map at the eye opening is likely to serve as substrate for the reorganization. Local horizontal connections in both in animals having organized orientation preference map as in cats and salt-and-pepper map as in mice are approximately isotropic (Burkhalter and Charles, 1990; Bosking et al., 1997; Buzás et al., 2006). Moreover, local connections in both cats (Martin and Schröder, 2013) and mice (Ko et al., 2011, 2013) more frequently link neurons with similar preferred orientations.

The mouse has emerged as an important model system for studying development of neural circuitry. This paper, to the best of our knowledge, presents for the first time a model on the development of receptive field for orientation selective cortical cells in layer IV in mouse V1. The model captures lack of clustering of similar (i) orientation selectivity preferences and (ii) OD in mice. Our study shows how the starting and closing time for critical period affect the orientation preference alignment between the two eyes and orientation tuning in cortical cells. The reaction diffusion model can be used to capture the formation of RF of orientation selective cells and their spatial organization in diverse animals such as mice or rats with no organized OR preference map to ferrets and cats with smooth OR preference map with pinwheels.

### Conflict of interest statement

The authors declare that the research was conducted in the absence of any commercial or financial relationships that could be construed as a potential conflict of interest.

## Supplementary material

Figure 1S **(A)** Synaptic weight development according to the LGN activity pattern when LGN cell at location J and its neighbors are active during weight update. **(B)** Synaptic weight development when LGN layer is activated by spontaneous activity pattern.
