# Supplementary figures and images for: Development and matching of binocular orientation preference in mouse V1
Source: Front Syst Neurosci. 2014 Jul 24;8:128. doi: 10.3389/fnsys.2014.00128 (PMC4109519; doi:10.3389/fnsys.2014.00128)

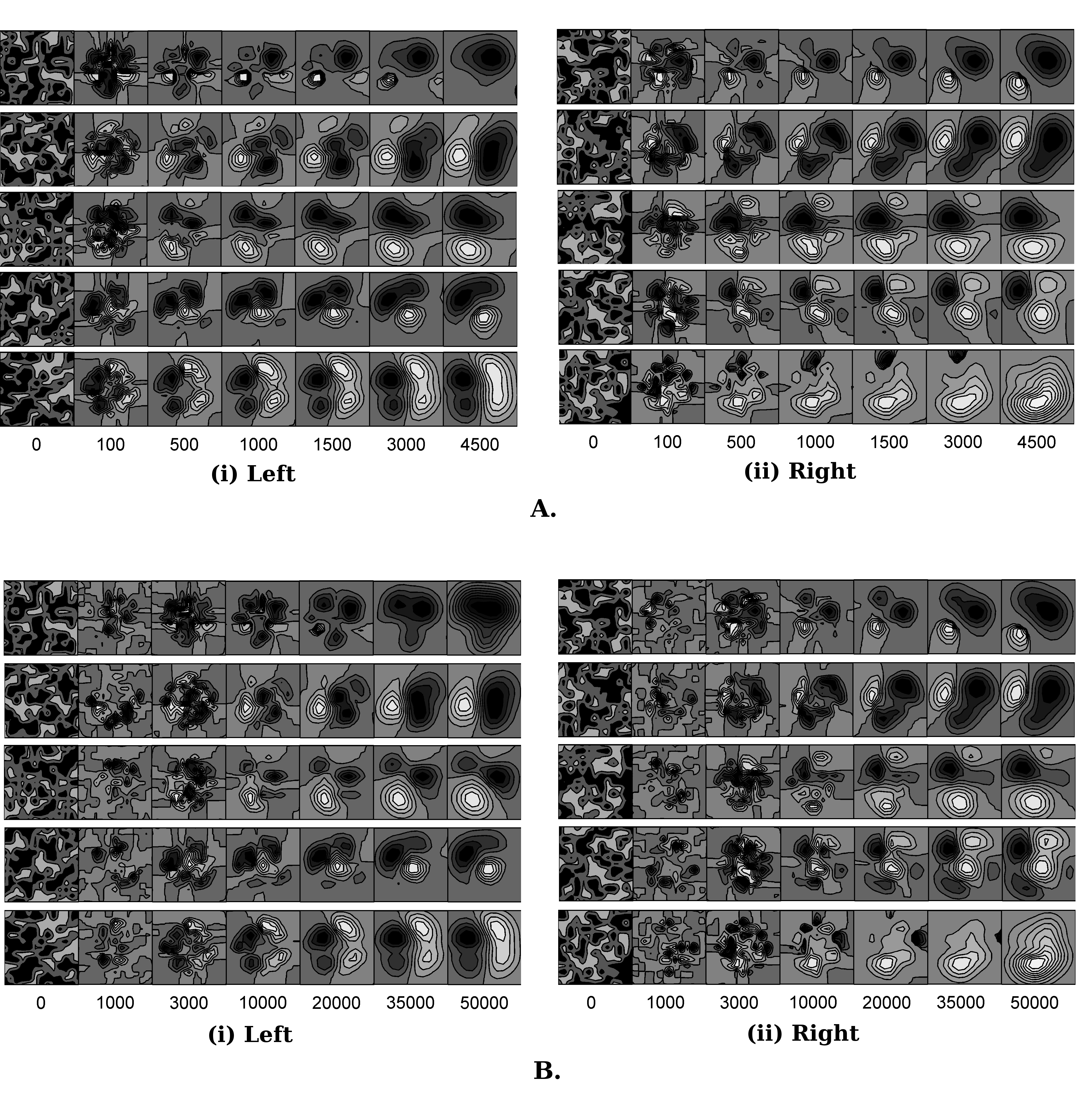

Supplement: Figure 1S — (A) Synaptic weight development according to the LGN activity pattern when LGN cell at location J and its neighbors are active during weight update. (B) Synaptic weight development when LGN layer is activated by spontaneous activity pattern. [file Image1.TIF]
