# Supplementary material for: Development and matching of binocular orientation preference in mouse V1
Source: Front Syst Neurosci. 2014 Jul 24;8:128. doi: 10.3389/fnsys.2014.00128 (PMC4109519; doi:10.3389/fnsys.2014.00128)
Supplement: Supplementary file 2 [file Presentation1.PDF]

Appendix

LGN model for Mouse

The spatial structure of the receptive fields for LGN cell for cat was modeled (Bhaumik and Mathur, 2003; Wehmeier et al., 1989; Wörgotter and Koch, 1991; Somers et al., 1995) using Difference of two Gaussians:

$G(x, y) = G_{center}(x, y) - G_{surround}(x, y)$ Where, $G(x, y) = \frac{K}{2\pi\sigma^2} e^{-\frac{x^2+y^2}{2\sigma^2}}$

We use the same model for LGN cells in mouse, but use mouse LGN data recorded by (Grubb and Thompson, 2003) to obtain the parameters. The center and surround sizes as reported in (Grubb and Thompson, 2003) correspond to σ_{center} = 238' and $\sigma_{surround}$ = 720'. K_{cen}/K_{surr} is set to 21/20 and K_{surr} is set to 1.15 and baseline spike rate of LGN of ~3.2 spikes /sec.

The spatial receptive field size of LGN cells is larger in mouse compared to cat. In cat σ_{center} = 10.6' and $\sigma_{surround}$ = 31.8' (Bhaumik and Mathur, 2003; Wehmeier et al., 1989; Wörgotter and Koch, 1991; Somers et al., 1995).

The Difference of Gaussian structure gives the LGN cells a band pass filtered structure. The temporal response for each Gaussian is given by:

$$H(t) = -\frac{t}{\tau}\exp(-\frac{t}{\tau})$$

Here, τ_{cen} = 6ms and τ_{surr} =12ms. The time constants for mouse are smaller than those for cat. In cat τ_{cen} = 10ms and τ_{surr} = 20ms. The smaller time constants in mouse correspond to higher optimal temporal frequency around 4Hz in mouse (Grubb and Thompson, 2003) as compared to 0.4 Hz in cat.

The spatio-temporal structure of each Gaussian is the product of spatial and temporal structures. Hence, $R_{center}(x, y, t) = G_{center}(x, y, t)\, H_{center}(x, y, t)$

The response of each Gaussian to a stimulus is calculated by convolution of filter structure with stimulus $I(x, y, t)$. The convolution is evaluated by multiplying the stimulus values with receptive field weights at stimulus grid points. Between successive LGN centers, there are 5 stimulus grids with separation between successive stimulus grids being 10'.

Finally, responses for ON center and OFF center LGN cells is calculated as:

$$ON(x, y, t) = [R_{center}(x, y, t) - R_{surround}(x, y, t)]^{+}$$

$$OFF(x, y, t) = [(2 \times \text{baseline}) - R_{center}(x, y, t) + R_{surround}(x, y, t)]^{+}$$

Baseline is activity of ON (OFF) cells to uniform background stimulus. K_{surr} and baseline (Somers et al., 1995) are adjusted to get spontaneous activity of ~3.2 spikes/sec and average spike rate of ~36 spikes/sec across all cells in ON and OFF cells when stimulus is presented.

Mouse shows poor contrast tuning (Grubb and Thompson, 2003) compared to cat. 90% contrast is selected in most experiments in mouse as compared to 50% contrast in cat experiments. We choose 90% contrast for the input stimulus.

Binary response of the LGN cells is produced by probabilistically generating spikes. The probability that an LGN cell fires a spike in interval t to t+ Δt (Δt <<1ms) is given by:

$$\mathrm{p}(\mathrm{x}, \mathrm{y}, \mathrm{t}) = \mathrm{p}_0 \times \Delta\mathrm{t} \times \mathrm{LGN}(\mathrm{x}, \mathrm{y}, \mathrm{t})$$

where p_0 is normalization constant.

LGN Activity

For development of orientation selectivity normal patterns of activity within both ON- and OFF-center pathways must be present (Chapman, 2000; Cang et al., 2005; Ackman et al., 2012). Pharmacological blockade of ON-LGN activity during development prevented development of orientation selectivity (Chapman and Gödecke , 2000). This suggests that LGN activity plays a role in development of orientation selectivity. Synchronous bursts of action potentials within all LGN layers in awake behaving ferrets prior to eye opening were recorded in (Weliky and Katz, 1999). Weliky and Katz (1999) observed that the firing in (i) neurons within the same eye-specific and center-type LGN layer were most highly correlated, (ii) neurons within the same eye-specific but opposite center-type LGN layer were more weakly correlated, and (iii) neurons within different eye-specific LGN layers had the weakest, but still significant, correlations. While the spatial and temporal structure of retinal spontaneous activity in mouse retina (Kerschensteiner and Wong, 2008) is available, details regarding the spontaneous neural activity within the next stage of the developing visual pathway, the LGN, is still unknown.

To study the effect of LGN activity on RF development till eye opening we have recast the synaptic weight development equation (1) as follows

$$\frac{\partial W_{IJ}^{l}}{\partial t} = \left(\gamma_1^l - K_{1J}^l\right)\left(\gamma_2 - K_{2I}^l\right) A_R(I,J)\, A_J^l\, W_{IJ}^l + \left(\frac{1}{(n+1)}\left(\sum_K A_K^l + A_J^l\right)\right) D_L(t) \nabla_J^2 W_{IJ}^l \qquad (4)$$

where $A_K^l = 1$ when the LGN cell at location K in the neighborhood of LGN cell at J, is active. $A_K^l = 0$ otherwise. The LGN cell at location J has n near neighbor LGN cells. In our case n=8. Note that when LGN cell at location J and its eight neighboring LGN cells are active then $\left(\frac{1}{(n+1)}\left(\sum_K A_K^l + A_J^l\right)\right) = 1$ and equation (4) reduces to equation (1).

For developing RFs till eye opening, with LGN activity as input we have assumed that correlations in firing in neurons in the mouse LGN are similar to that in the Ferret. We have extended the single eye activity pattern as modeled by (Goodhill, 1993) to obtain activity pattern in left and right eye specific ON and OFF LGN layers. The details regarding the modeling of LGN activity before and after eye opening is given in the next section.

In Figure 1S we show RFs developed with LGN cell activities where, (i) LGN cell at J^{th} location and its eight neighboring LGN cells are active during weight update, and (ii) activities of LGN cell at location J and its eight neighboring LGN cells are determined by LGN activity pattern. The top row in Figure 1S shows the left and the right eye RFs developed using equation (1) and (2). The RFs at 500 epochs correspond to RFs at the start of the critical period. The bottom row in Figure 1S shows RFs developed using LGN activity before and after eye opening. The eye opening time corresponds as 4000 epoch. The RFs at 7000 epochs correspond to RFs at the start of critical period. For the synaptic weight development from 7000 epochs, subregion correspondence factor, C^l was included in the first term in equation (4) for development of RF during the critical period. The RFs were developed till 50000 epoch and are shown in the bottom row in Figure 1S.

Weliky and Katz (1997) had shown that development of orientation tuning gets disrupted when ON and OFF ganglion cells are synchronously activated. The artificially induced highly correlated activity between ON and OFF LGN inputs was thought to disrupt subfield segregation in simple cells (Weliky M, Katz, 1997; Weliky, 2000). We have modeled the activity pattern in different type of LGN layers as being un-correlated after eye opening. The activities in the same type of LGN layers in the two eyes are correlated.

Note that RFs developed using LGN activity are qualitatively similar to the RFs developed using equation (1) and (2), but require a much larger number of iterations. Firing activity in nearby LGN cells are correlated (Weliky and Katz, 1999). In our model the synaptic weight update at LGN location J depends on activity of LGN cell at location J and its eight nearest neighbors. Our study indicates that during the development the details regarding the activity pattern are not important, as long as all areas in LGN are activated equally overtime, so that RFs with well defined ON and OFF subregions develop. Independence from details in LGN activity pattern ensures robustness during development.

Spontaneous activity pattern in LGN

In this section, spontaneous activity in LGN used to generate weights in Figure 1S is described. The method for generating spontaneous activity used here is a modification of the method given in (Koulakov and Chklovskii, 2001) for generating activity for ON and OFF layers in one eye. Here, we have extend it for two eyes.

Let Q be a 4x4 matrix representing the correlation pattern across 4 layers (ON and OFF type layers across 2 eyes). First, 4 independent binary variables are sampled at a given location in LGN. These are represented as $P = [P_l^{ON}(i), P_l^{OFF}(i), P_r^{ON}(i), P_r^{OFF}(i)]^T$. Where $P_l^{ON}(i) \in \{-0.5, +0.5\}$ is the sampled value at ON-type cell in Left LGN at location i. The covariance of P is $0.25I$.

Using the Eigen value decomposition of $Q = U\sum U^T$, if we multiply P with $U\sum^{1/2}$, we get the desired correlation structure arross layers. So, $R = U\sum^{1/2}P$. Here R is the correlated samples at location I across the 4 layers and $R = [R_l^{ON}(i), R_l^{OFF}(i), R_r^{ON}(i), R_r^{OFF}(i)]^T$
Afterwards, spatial correlation between activities of neighbouring cells in a layer is introduced. This is done by convolving each $R_L^{ON}(i)$ with a gaussian $G(i) = \exp(\frac{-i^2}{\sigma^2})$. σ = 125 is used. This

is done for other layers also. This results in spatially smooth spontaneous activity patterns i.e., ON cells of an eye tend to fire together with ON cells at short distances in the same eye. Similarly for OFF cells also.

Finally, the activity for cells is generated as follows (for ON-type LGN in Left eye) :

$$A_l^{ON}(i) = \begin{cases} 1 \; if \; new \; R_l^{ON}(i) > \theta \\ 0 \quad otherwise \end{cases}$$

θ = 0.0 is used in our case. We have similarly obtained $A_l^{OFF}(i)$, $A_r^{ON}(i)$ and $A_r^{OFF}(i)$.

For Simulations in Figure 1S, a new activity pattern is generated at every epoch or iteration. 0th epoch in our simulation corresponds to beginning of development of orientation selectivity at P10 in mouse. From 0th epoch till 4000th epochs, the correlation structure is taken as –

$$Q = \begin{bmatrix} 1 & 0.48 & 0.248 & 0.24 \\ 0.48 & 1 & 0.24 & 0.248 \\ 0.248 & 0.24 & 1 & 0.48 \\ 0.24 & 0.248 & 0.48 & 1 \end{bmatrix}$$

Where the order of layers is: [Left ON, Left OFF, Right ON, Right OFF]. 4000th epoch correspond to eye opening at P15. The correlations between the ON and OFF layers of an eye in mouse are taken from (Bonin et al., 2011). The correlation between layers of same type (ON-ON or OFF-OFF) across eyes is taken from (Hagihara and Ohki, 2013).

The spontaneous LGN activities generated and used for RF development till eye opening in this paper have: (i) Near neighbour correlation in a layer: ~34.83 %, (ii) Left ON- Left OFF: 33.77 % correlation, (iii)Left ON - Right ON: 18.46% correlation, and (iv)Left ON- Right OFF: 18.35% correlation .

After eye opens are P15, (4000 epochs), the activity pattern across ON and OFF layers of an eye become un-correlated and that of layers of same type (both ON-type or both OFF-type) across two eyes become perfectly correlated. Hence,

$$Q = \begin{bmatrix} 1 & 0 & 1 & 0 \\ 0 & 1 & 0 & 1 \\ 1 & 0 & 1 & 0 \\ 0 & 1 & 0 & 1 \end{bmatrix}$$

The spontaneous LGN activities generated and used for RF development after eye opening have: (i)Near neighbour correlation: ~33.47% correlation, (ii) Left on- Left Off : 0.27 % correlation, (iii) Left on - Right on:100% correlation, and (iv) Left on- Right off : 0.27% correlation.

In the simulations using activity pattern after eye opening C-iter is taken as 7000 and end of critical period is 50000th epochs. The number of epochs scale nearly 10 times as compared to numbers required in simulation when all cells are assumed active all the time.
